# Supplementary material for: Wnt/β-Catenin-Signaling Modulates Megakaryopoiesis at the Megakaryocyte-Erythrocyte Progenitor Stage in the Hematopoietic System
Source: Cells. 2023 Dec 4;12(23):2765. doi: 10.3390/cells12232765 (PMC10706863; doi:10.3390/cells12232765)

Article

Wnt/β-Catenin-Signaling Modulates Megakaryopoiesis at the Megakaryocyte-Erythrocyte Progenitor Stage in the Hematopoietic System

Burak H. Yalcin [1,†], Jadranka Macas [1], Eliza Wiercinska [2], Patrick N. Harter [1,‡], Malak Fawaz [3], Tessa Schmachtel [3], Ilaria Ghiro [1,‡], Ewa Bieniek [4], Djuro Kosanovic [4,§], Sonja Thom [1], Marcus Fruttiger [5], Makoto M. Taketo [6], Ralph T. Schermuly [4], Michael A. Rieger [3,7,8,9], Karl H. Plate [1,8,10], Halvard Bonig [2,11] and Stefan Liebner [1,9,10,*]

[1] Institute of Neurology (Edinger Institute), University Hospital Frankfurt, Goethe University,
60590 Frankfurt am Main, Germany; burakyalcin@gmx.de (B.H.Y.); Macas@em.uni-frankfurt.de (J.M.);
patrick.harter@med.uni-muenchen.de (P.N.H.); ilariaghiro@icloud.com (I.G.); thom@med.uni-frankfurt.de (S.T.); plate@em.uni-frankfurt.de (K.H.P.)

[2] Institute for Transfusion Medicine and Immunohaematology, Goethe University Frankfurt,
and DRK-Blutspendedienst BaWüHe, 60528 Frankfurt am Main, Germany; e.wiercinska@blutspende.de (E.W.); h.boenig@blutspende.de (H.-B.B.)

[3] Department of Medicine, Hematology/Oncology, University Hospital Frankfurt,Goethe University, 60590 Frankfurt am Main, Germany; m.fawaz@hotmail.de (M.F.); schmachtel@med.uni-frankfurt.de (T.S.); m.rieger@em.uni-frankfurt.de (M.R.)

[4] German Center for Lung Research (DZL), Department of Internal Medicine, Excellence Cluster
Cardio-Pulmonary Institute (CPI), Justus Liebig University of Giessen, Aulweg 130, 35392 Giessen, Germany; ewa.bieniek@innere.med.uni-giessen.de (E.B.); djurokos13@gmail.com (D.K.); ralph.schermuly@innere.med.uni-giessen.de (R.T.S.)

[5] UCL Institute of Ophthalmology, London EC1V 9EL, UK; m.fruttiger@ucl.ac.uk

[6] Kyoto University Hospital-iACT Graduate School of Medicine, Kyoto University, Kyoto 06-8501, Japan; taketou.makoto.52x@st.kyoto-u.ac.jp

[7] German Cancer Consortium (DKTK) at the German Cancer Research Center, 69120 Heidelberg, Germany

[8] Frankfurt Cancer Institute (FCI), 60596 Frankfurt am Main, Germany

[9] Excellence Cluster Cardio-Pulmonary Institute (CPI), Partner site Frankfurt, 60590 Frankfurt am Main, Germany

[10] German Center for Cardiovascular Research (DZHK), Partner site Frankfurt/Mainz, 60590 Frankfurt am Main, Germany

[11] Department of Medicine/Division of Hematology, University of Washington, Seattle, WA 98195, USA

* Correspondence: stefan.liebner@kgu.de; Tel.: +49-(0)69-6301-84154

† Current address: Abbott GmbH, Max-Planck-Ring 2, 65205 Wiesbaden, Germany.

‡ Current address: Radiation Biology and DNA Repair, Darmstadt University of Technology,
64289 Darmstadt, Germany.

§ Current address: Department of Pulmonology, Sechenov First Moscow State Medical University (Sechenov University), Moscow 119992, Russia.

Citation: Yalcin, B.H.; Macas, J.; Wiercinska, E.; Harter, P.N.; Fawaz, M.; Schmachtel, T.; Ghiro, I.; Bieniek, E.; Kosanovic, D.; Thom, S.; et al. Wnt/β-Catenin-Signaling Modulates Megakaryopoiesis at the Megakaryocyte–Erythrocyte Progenitor Stage in the Hematopoietic System. *Cells* **2023**, *12*, x. https://doi.org/10.3390/xxxxx

Academic Editor: Katya Ravid

Received: 11 September 2023
Revised: 20 November 2023
Accepted: 29 November 2023
Published: date

Copyright: © 2023 by the authors. Submitted for possible open access publication under the terms and conditions of the Creative Commons Attribution (CC BY) license (https://creativecommons.org/licenses/by/4.0/).

Supplementary Figure Legends

Suppl. Fig. 1:

Exemplary gating strategy for leukocytes. Single (A2), debris free (A3) blood cells were counted using leukocyte-specific markers such as Ly6G for granulocytes or F4/80 for monocytes. For the lymphoid compartment $CD4^+$ T cells or $CD19^+$ B cells were quantified. $1x10^5$ events were counted and analyzed using FACS Canto TM II.

Suppl. Fig. 2:

Gating of GFP^+ cells from MKPs in BM recipients after tamoxifen treatment. (A) Flow-cytometry gating strategy for the detection of MKPs in BM cells. In first line, BM cells (A1) were gated for CD150 (A2) and then gated for $CD41^-$ (A3) and $CD41^+$ (A4). $CD41^+$ cells were gated for GFP (A5), showing 91.0% of all $CD41^+$ cells. These cells exhibit high granularity and size (A6). $1x10^6$ were counted on the FACS CantoTM II.

(B) FMO control gating for GFP expressing cells from BM. $1x10^6$ were counted on the FACS CantoTM II.

Suppl. Fig. 3:

Flow-cytometry gating strategy for analyzing BM engraftment in recipient mice from peripheral blood. The blood cells (A1) were gated for the newly engrafted $CD45.2^+$ cells (A3) after irradiation and the pre-existing recipient $CD45.1^+$ cells (A2) after irradiation and BMT. To track the hematopoiesis of mature blood cell types by newly engrafted HSCs, the blood cells (A1) were gated for $CD19^+$ B cells (A4), $CD3^+$ T cells (A5) and $CD11b^+$ (A6) cells in combination with the engraftment indicator markers CD45.1 and CD45.2 (A4.1, A5.1, A5.2, A6.1, A6.2). $1x10^5$ events were counted on the FACS Canto TMII.

Suppl. Fig. 4:

Transgene GFP-reporter analysis of MKPs in BMT recipients after tamoxifen treatment. (A) Flow-cytometry gating strategy for the detection of MKPs in BMT animals. In first line, BM cells (A1) were checked whether they have origin of donor (A2) or recipient mice (A3). MKPs (A4, A5) were gated out of $CD45.2^+$ donor cells and plotted for GFP presence (A6). $1x10^6$ were counted on the FACS CantoTM II.

(B) Gated MKPs (A4) did rise from newly engrafted donor BM (CD45.2+). BMT CTL MKPs were not expressing the transgene GFP-reporter (red box), whereas the mutants ($Ctnnb1^{BM-GOFwt/fl}$) expressed the GFP-reporter (green box).

Suppl. Fig. 5:

Analysis of the skin from $Ctnnb1^{BM-GOFwt/fl}$ and $Ctnnb1^{BM-CTL}$ mice. (A) HE staining on skin cryosection samples revealed normal formation of hair follicles (asterisk) in $Ctnnb1^{BM-CTL}$ animals. In contrast, $Ctnnb1^{BM-GOFwt/fl}$ mice developed hyperkeratosis with abnormalities in the formation of hair follicles (asterisk). Scale bar: 250µm

(B) KI67 staining on paraffin embedded skin sections. In $Ctnnb1^{BM-CTL}$ animals, the KI67 staining occurred mainly in hair follicles (asterisk) and at lower level in the epidermal layer (arrowheads). In the $Ctnnb1^{BM-GOFwt/fl}$ samples, epidermal cells were strongly positive for Ki67 (arrowheads). Furthermore, hyperplastic hair follicles (asterisk) were strongly Ki67 positive.

The dermal layer showed several individual stained parenchymal cells (arrows). Scale bar: 250 µm

Suppl. Fig. 6:
Analysis of lung tissue from Ctnnb1$^{BM\text{-}GOFwt/fl}$ and Ctnnb1$^{BM\text{-}CTL}$ mice. (A)Analysis of lung cryosections using different staining methods: there was no apparent difference between Ctnnb1$^{BM\text{-}CTL}$ and Ctnnb1$^{BM\text{-}GOFwt/fl}$ animals with regard to the parenchyma of the lung (HE staining). (B) Furthermore, no anomalies could be seen in the collagen content (Picro-Sirius staining) or (C) in the inflammation status by the staining of T cells (CD3, arrows in CTL and Ctnnb1$^{BM\text{-}GOFwt/fl}$. Scale bar: 50µm

Suppl. Fig. 7:
CBC assay from PB from Ctnnb1$^{BM\text{-}GOFwt/fl}$ and Ctnnb1$^{BM\text{-}CTL}$ mice, showing hemoglobin (Hb, g/dL), hematocrit (HCT, %), mean corpuscular volume (MCV, fL), mean corpuscular hemoglobin (MCH, pg), mean corpuscular hemoglobin concentration (MCHC, g/dL), red cell distribution width (RDW, %) for of CTL (n=12), Ctnnb1$^{BM\text{-}GOFw/fl}$ (n=6) and Ctnnb1$^{BM\text{-}GOFfl/fl}$ (n=7) mice.

Suppl. Fig. 8:
Blood analysis by FACS from PB after BMT from Ctnnb1$^{BM\text{-}GOFwt/fl}$ and Ctnnb1$^{BM\text{-}CTL}$ mice. (A-D) BM engraftment analysis in PB cells. Using the gating strategy shown in Fig. 4 allowed to control for lineage specific engraftment, such as myeloid cells and lymphoid cells (T cells and B cells). The BMT was successful since most PB cells had CD45.2 positive origins. n=3
(E-G) CBC assay analysis of BMT (peripheral blood) PB samples. There were no significant alterations in the erythroid compartment or the thrombocytes. However, erythrocytes in BMT_Ctnnb1$^{BM\text{-}GOFwt/fl}$ were slightly reduced. The WBCs were decreased in BMT_Ctnnb1$^{BM\text{-}GOFwt/fl}$ but remaining in the normal range though. Normal range WBCs: 1.810.7 K/µl, normal range erythrocytes: 6.36-9.42 M/µl, normal range thrombocytes: 592-2972 K/µl.

Suppl. Fig. 9:
Analysis of HSCs and Progenitors in the BM of BMT animals. After the successful engraftment of newly transplanted BM and tamoxifen application, HSCs and several hematopoietic progenitors were counted using FACS. The activation of the Wnt/β-catenin pathway in BMT Ctnnb1$^{BM\text{-}GOF}$ did not lead to changes in the differentiation of HSCs, CMPs, MEPs and GMPs in comparison to the CTL. Gating strategy shown in Suppl. Fig. 2 was applied and $1x10^6$ events were recorded.

Suppl. Fig. 10:
Ctnnb1$^{EC\text{-}GOFwt/fl}$ transgene analysis of MKPs in the BM after tamoxifen treatment. (A) Scheme of Cdh5(PAC)-CreERT2 mice crossed with Ctnnb1$^{lox(ex3)/(ex3)}$ mice to achieve endothelial-specific activation of β-catenin (Ctnnb1$^{EC\text{-}GOFwt/fl}$). (B) Flow-cytometry gating strategy for the detection of MKPs in BM of control (CTL) animals. At first, the BM was gated

for CD150$^+$ cells, followed by gating for the CD150$^+$ and CD41$^+$ double positive MKPs. 1x10^6 were counted on the FACS CantoTM II.

(C) Detection of CD150$^+$ and CD41$^+$ double positive MKPs in BM of Ctnnb1$^{EC\text{-}GOFwt/fl}$ animals. (D) Quantification of MKPs as % of bone marrow (BM) cells. (E) Fluorescence minus one (FMO) control for CD41-PE/Cy7. (F) Fluorescence minus one (FMO) control for CD150-PE.

Suppl. Fig. 11:

Ctnnb1$^{EC\text{-}GOFwt/fl}$ transgene analysis of MKPs in the spleen after tamoxifen treatment. (A) Flow-cytometry gating strategy for the detection of MKPs in spleen of control (CTL) animals. At first, the BM was gated for CD150$^+$ cells, followed by gating for the CD150$^+$ and CD41$^+$ double positive MKPs. 1x10^6 were counted on the FACS CantoTM II.

(B) Detection of CD150$^+$ and CD41$^+$ double positive MKPs in the spleen of Ctnnb1$^{EC\text{-}GOFwt/fl}$ animals. (C) Quantification of MKPs as % of spleen (SP) cells. (D) Fluorescence minus one (FMO) control for CD41-PE/Cy7. (E) Fluorescence minus one (FMO) control for CD150-PE.

Suppl. Fig. 12:

Ctnnb1$^{EC\text{-}GOFwt/fl}$ transgene is recombined and Wnt/β-catenin target is activated upon 4-OHT treatment in vitro.

(A) Isolated bone marrow mononucleated cells from PDGFb-iCreERT2$^{wt/wt}$:Ctnnb1$^{wt/lox(Ex3)}$ (Ctrl) PDGFb-iCreERT2$^{wt/cre}$:Ctnnb1$^{wt/lox(Ex3)}$ (GOF) mice were cultured for 7 days in StemSpanTM SFEM media (STEMCELL #09650) with stem cell factor (m-SCF, 100 ng/ml) and thrombopoietin (TPO, 100 ng/ml), treated every second day with 25 µM 4-hydroxy-tamoxifen (4-OHT) and 7 days.

(B) Pdgfb-expressing brain microvascular endothelial cells (MBMECs) from the same mice were isolated, cultured and harvested after 7 days.

From both cells types gDNA and total RNA was isolated and recombination of the transgen and induction of the Wnt/β-catenin target Axin2 was analyzed by PCR and qRT-PCR, respectively (n=1). The data confirming recombination as well as Axin2 induction in GOF cells.

Suppl. Fig. 1, Yalcin et al.

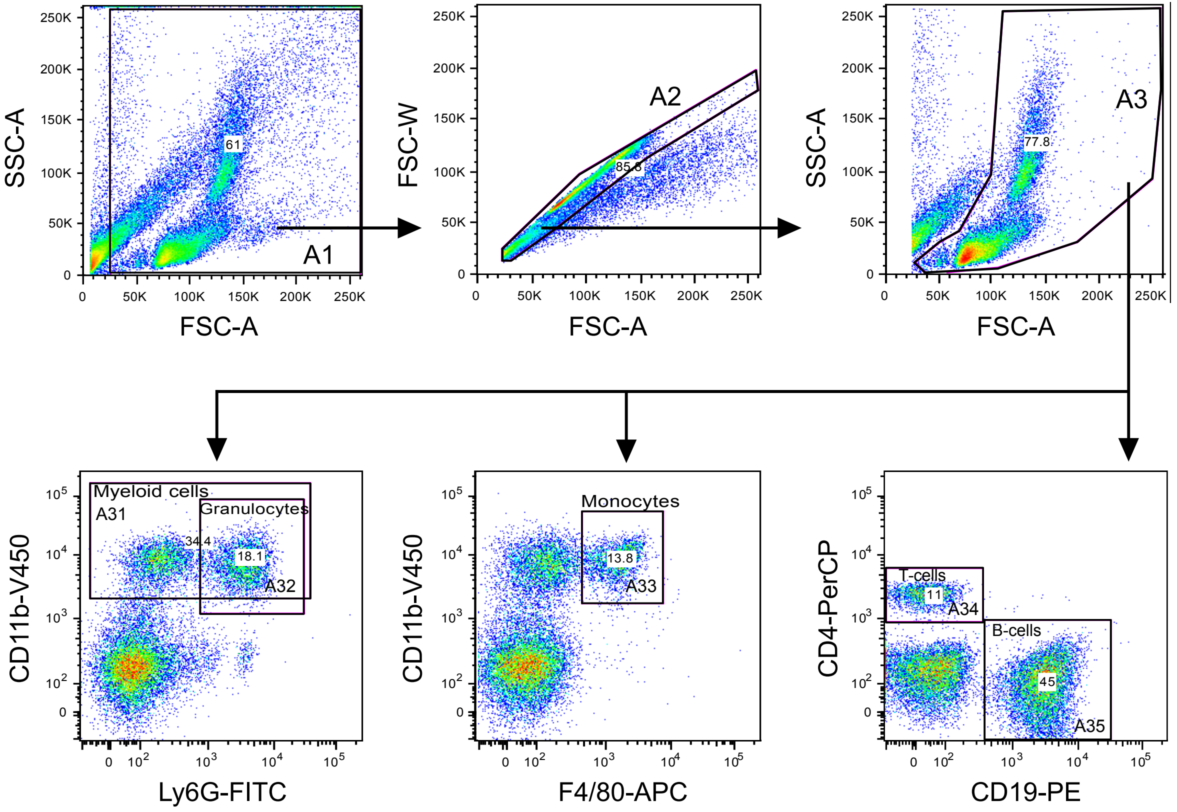

Suppl. Figure 2, Yalcin et al.

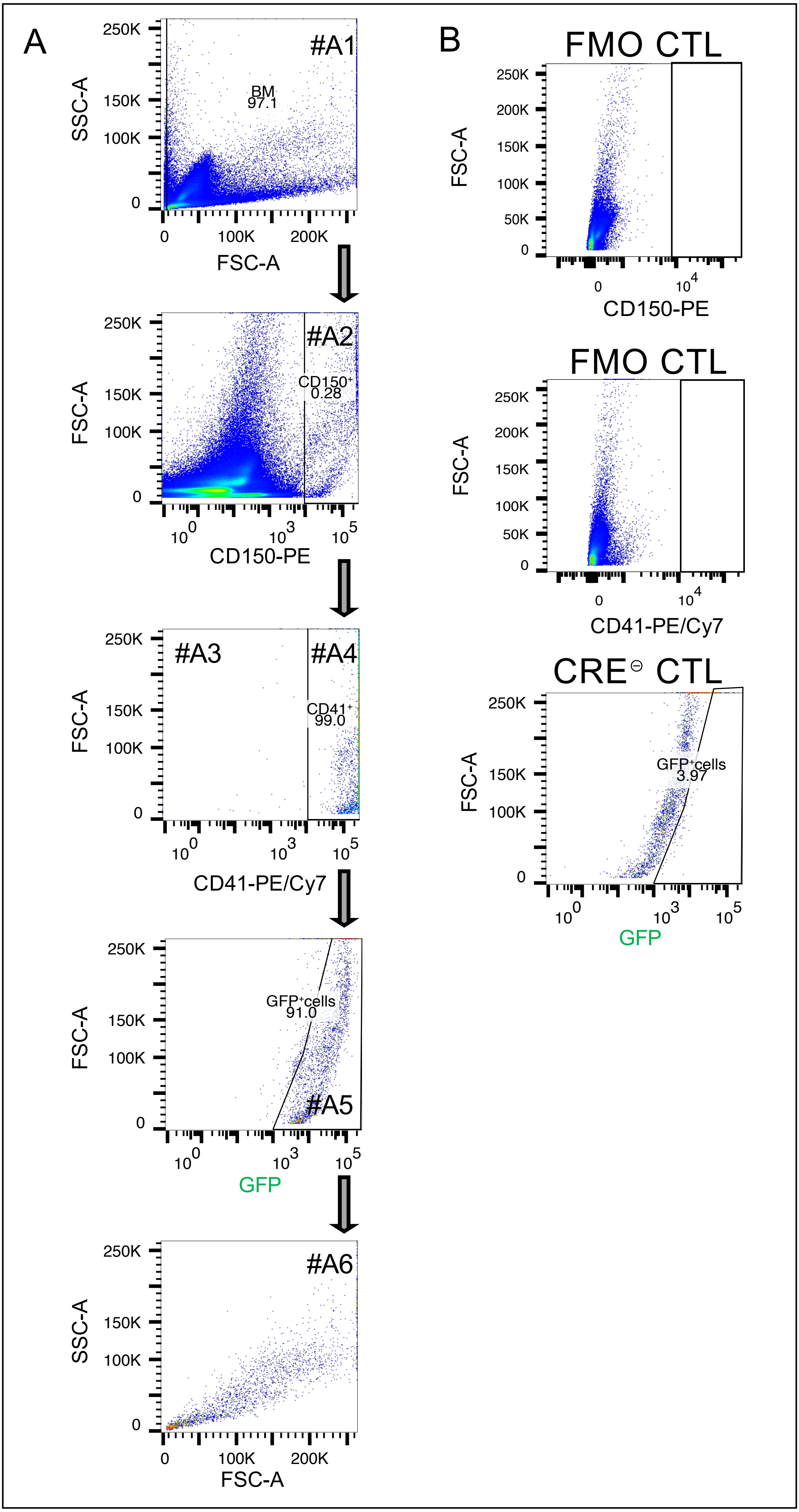

Suppl. Fig. 3, Yalcin et al.

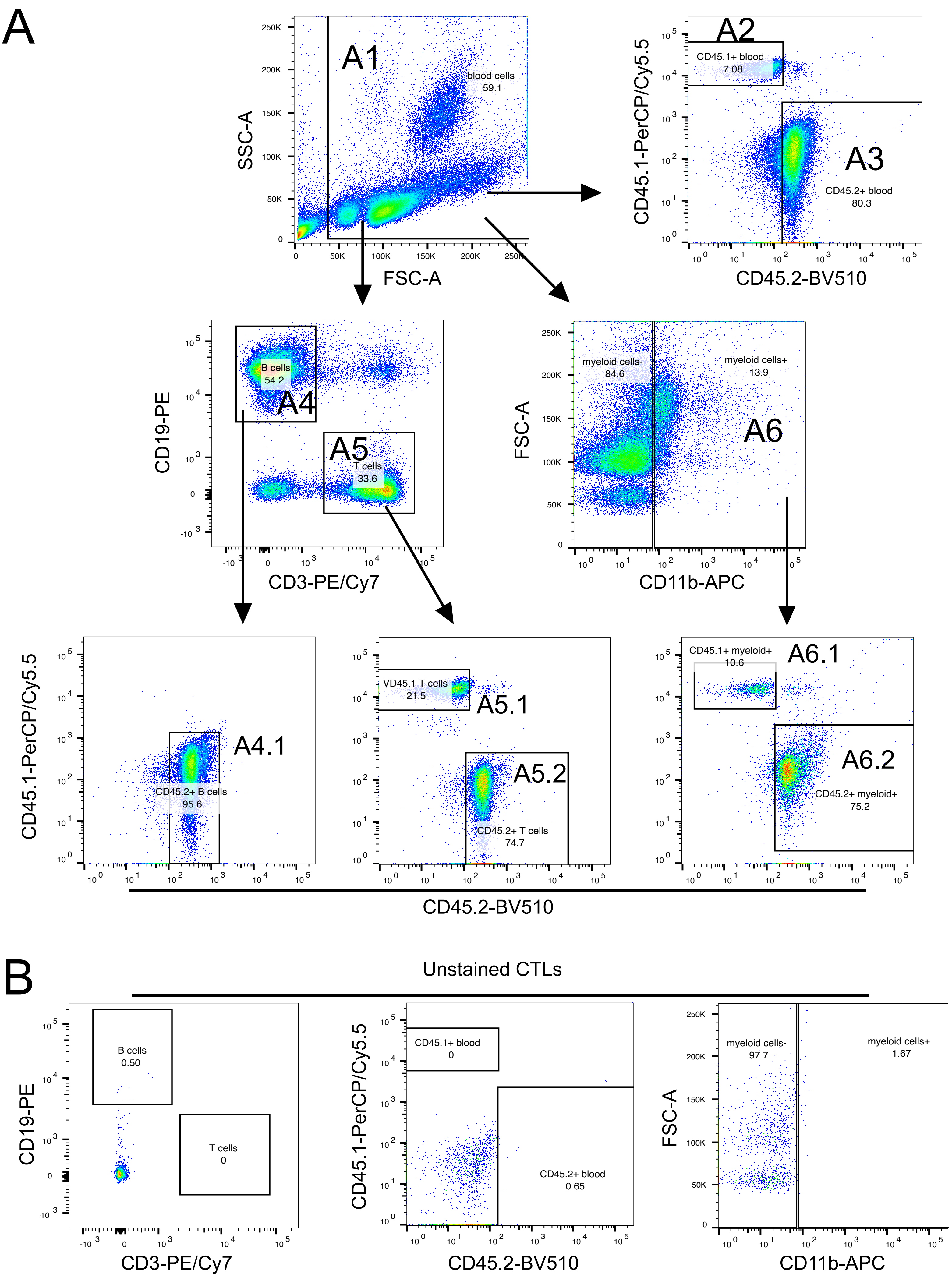

Suppl. Fig. 4, Yalcin et al.

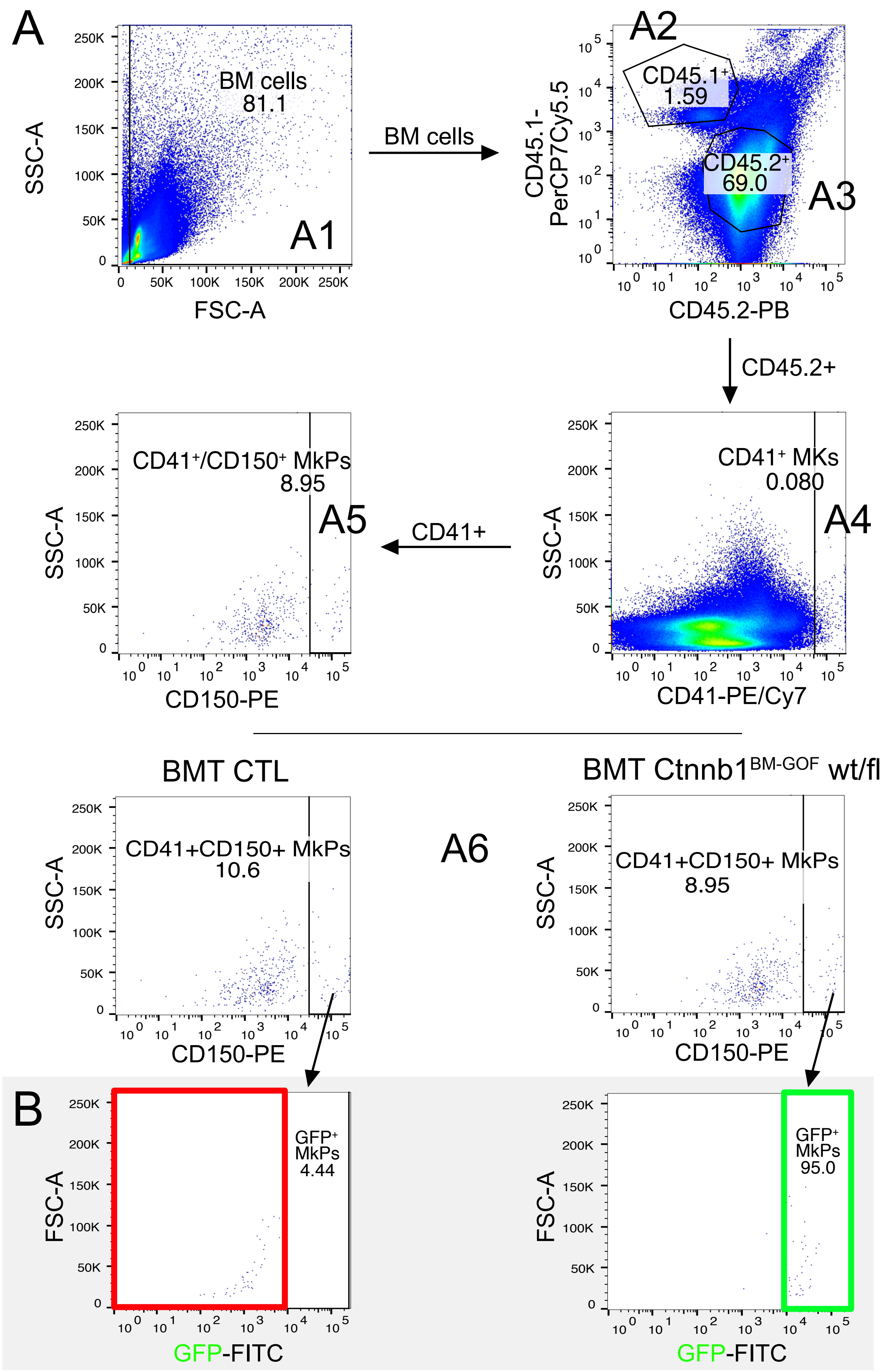

Suppl. Fig. 5, Yalcin et al.

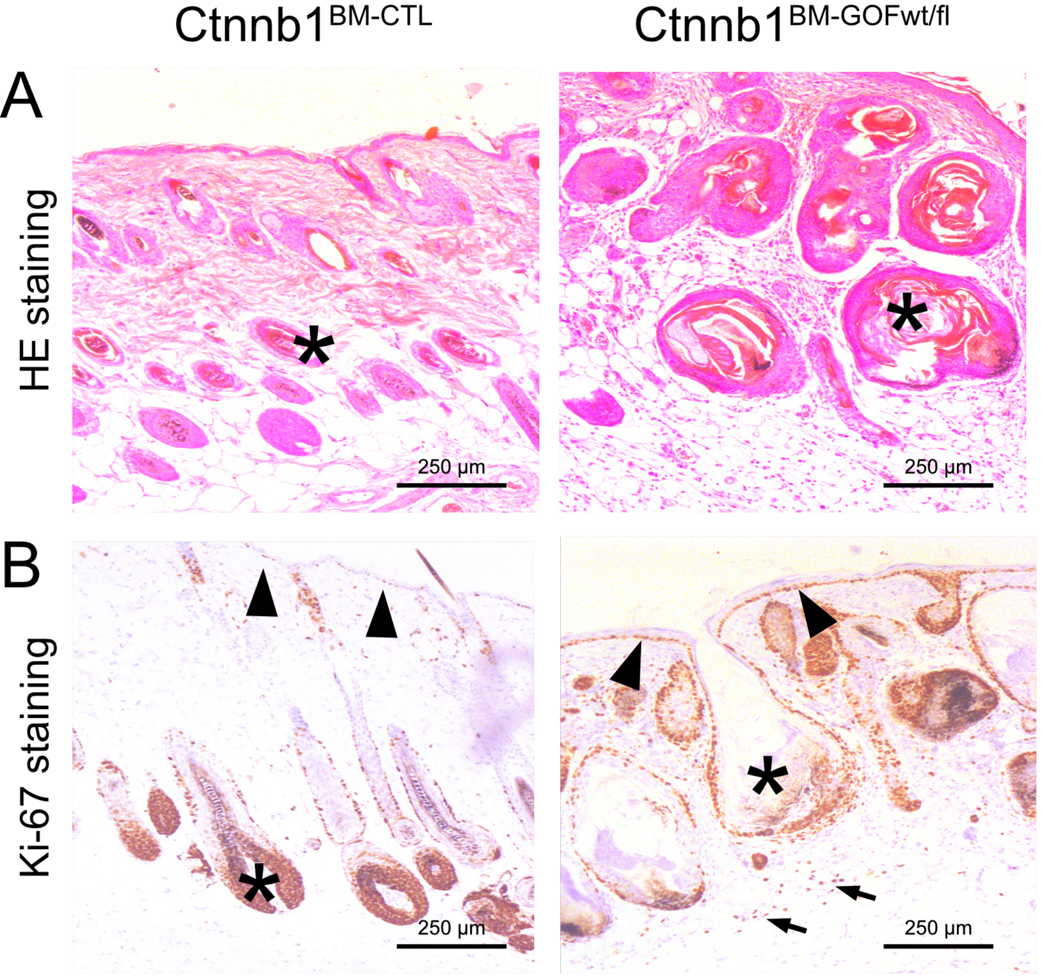

Suppl. Fig. 6, Yalcin et al.

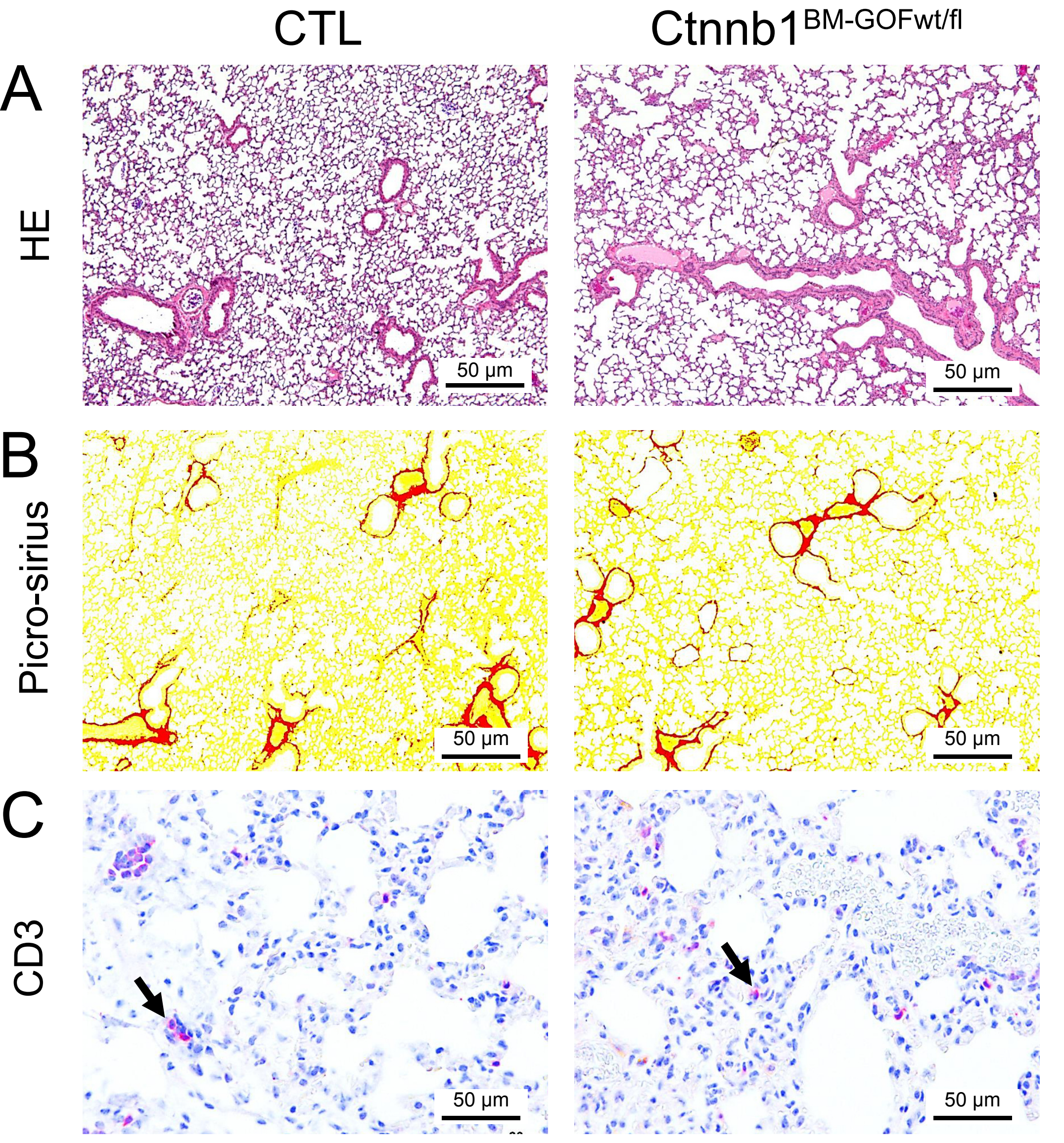

Suppl. Figure 7, Yalcin et al.

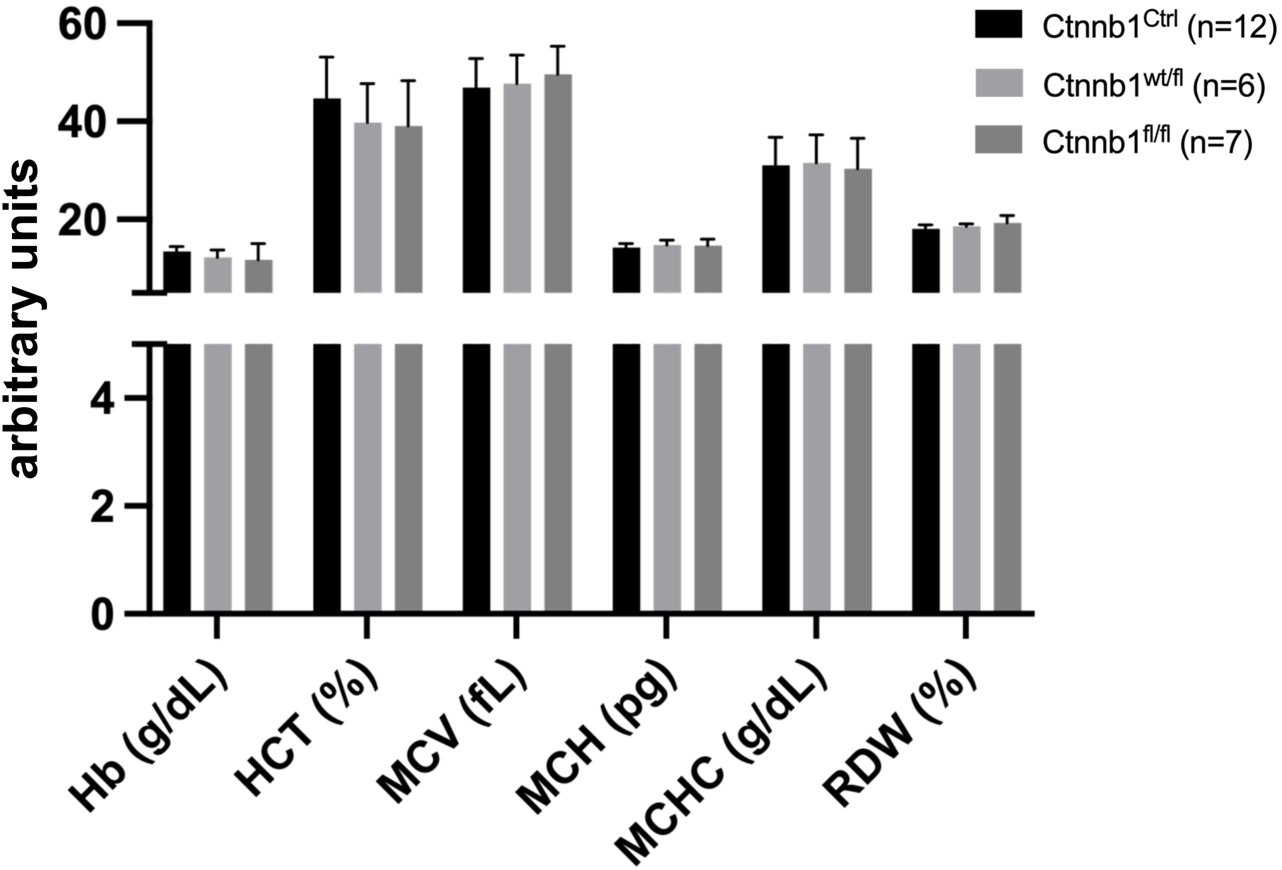

Suppl. Figure 8, Yalcin et al.

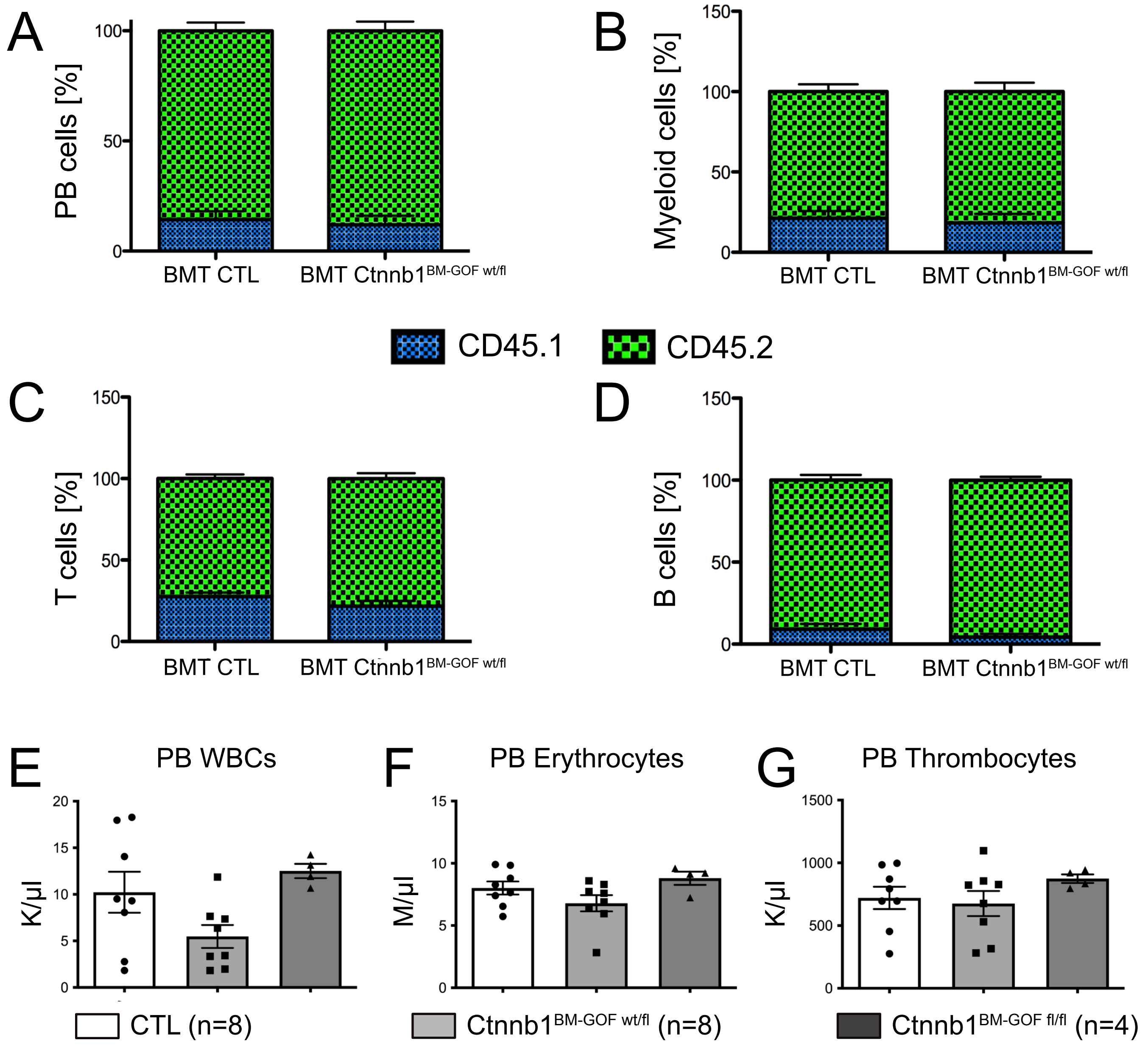

Suppl. Fig. 9, Yalcin et al.

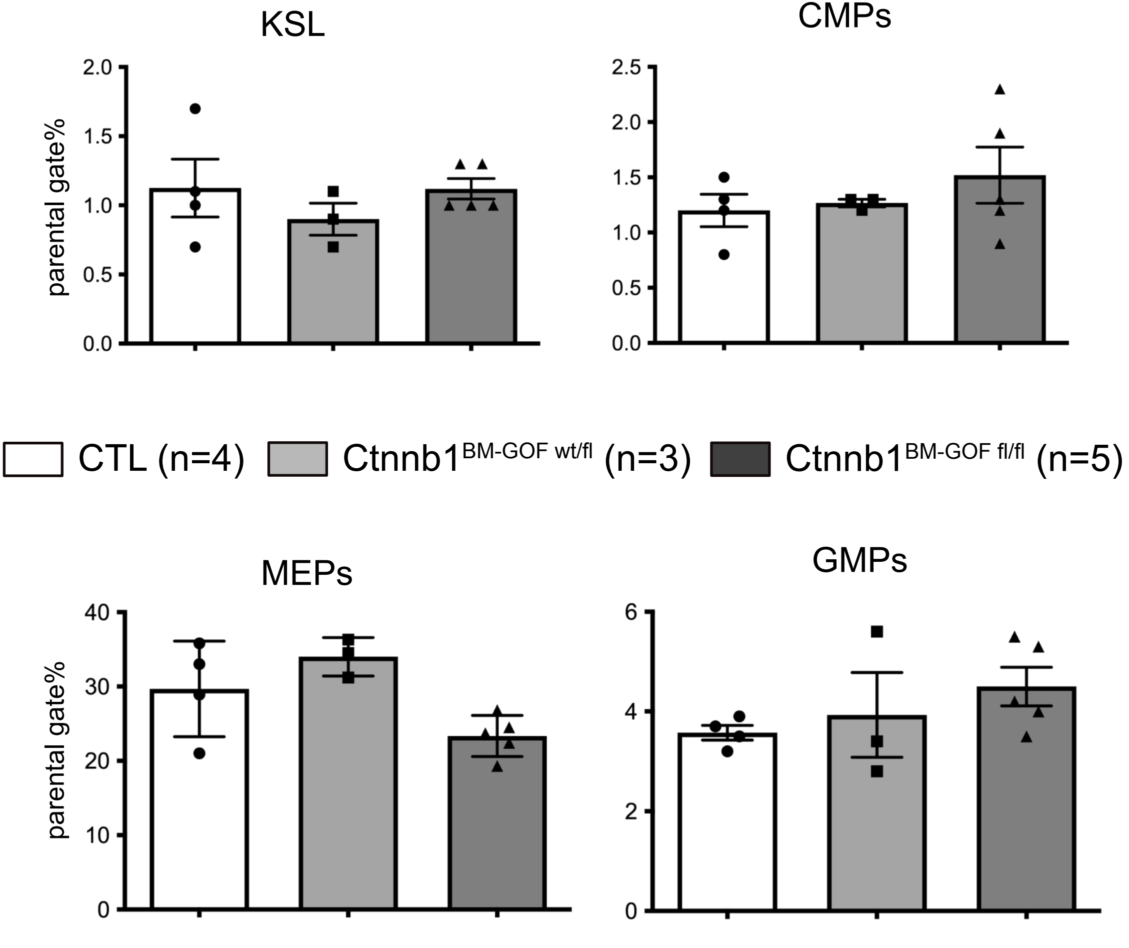

Suppl. Fig. 10, Yalcin et al.

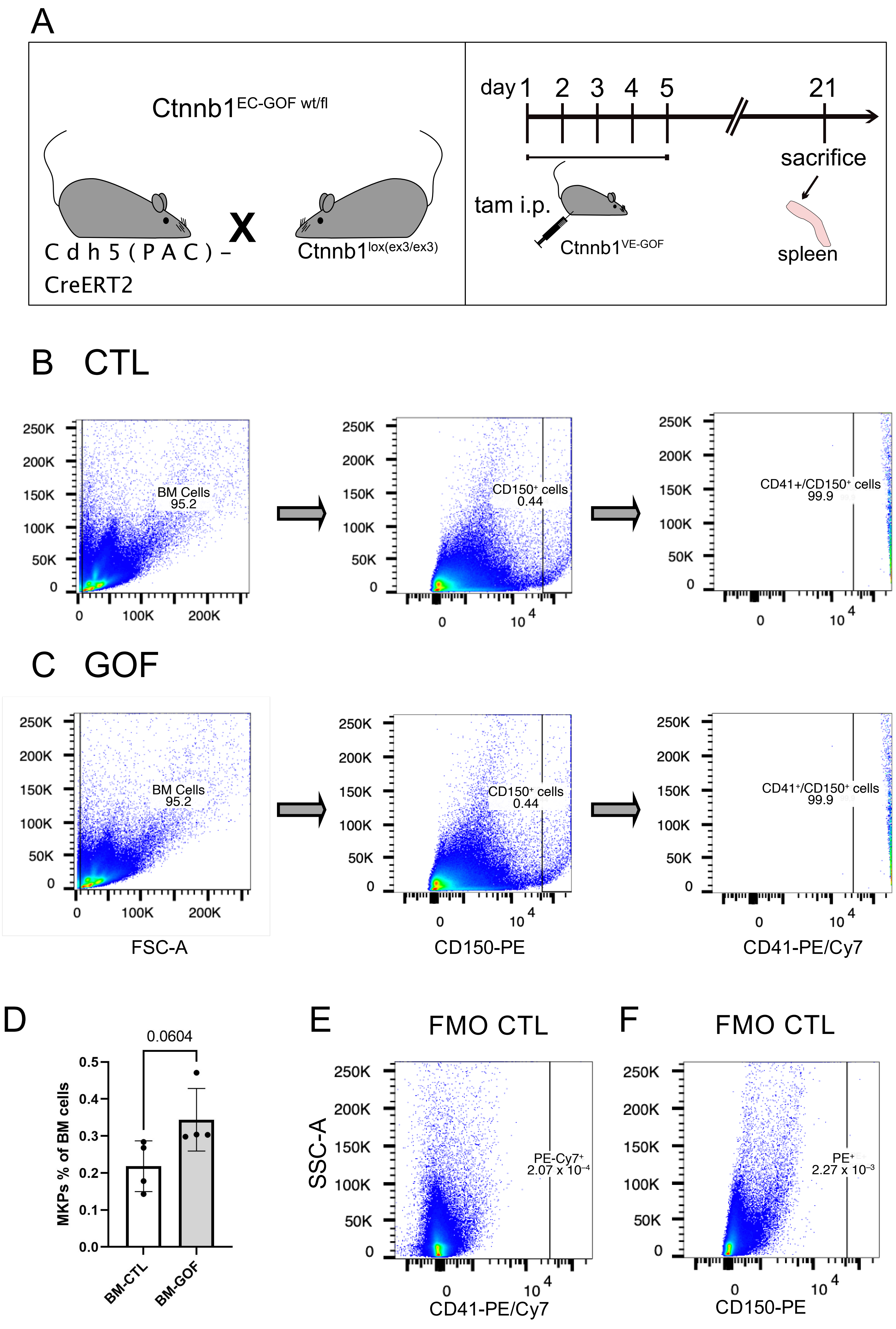

Suppl. Fig. 11, Yalcin et al.

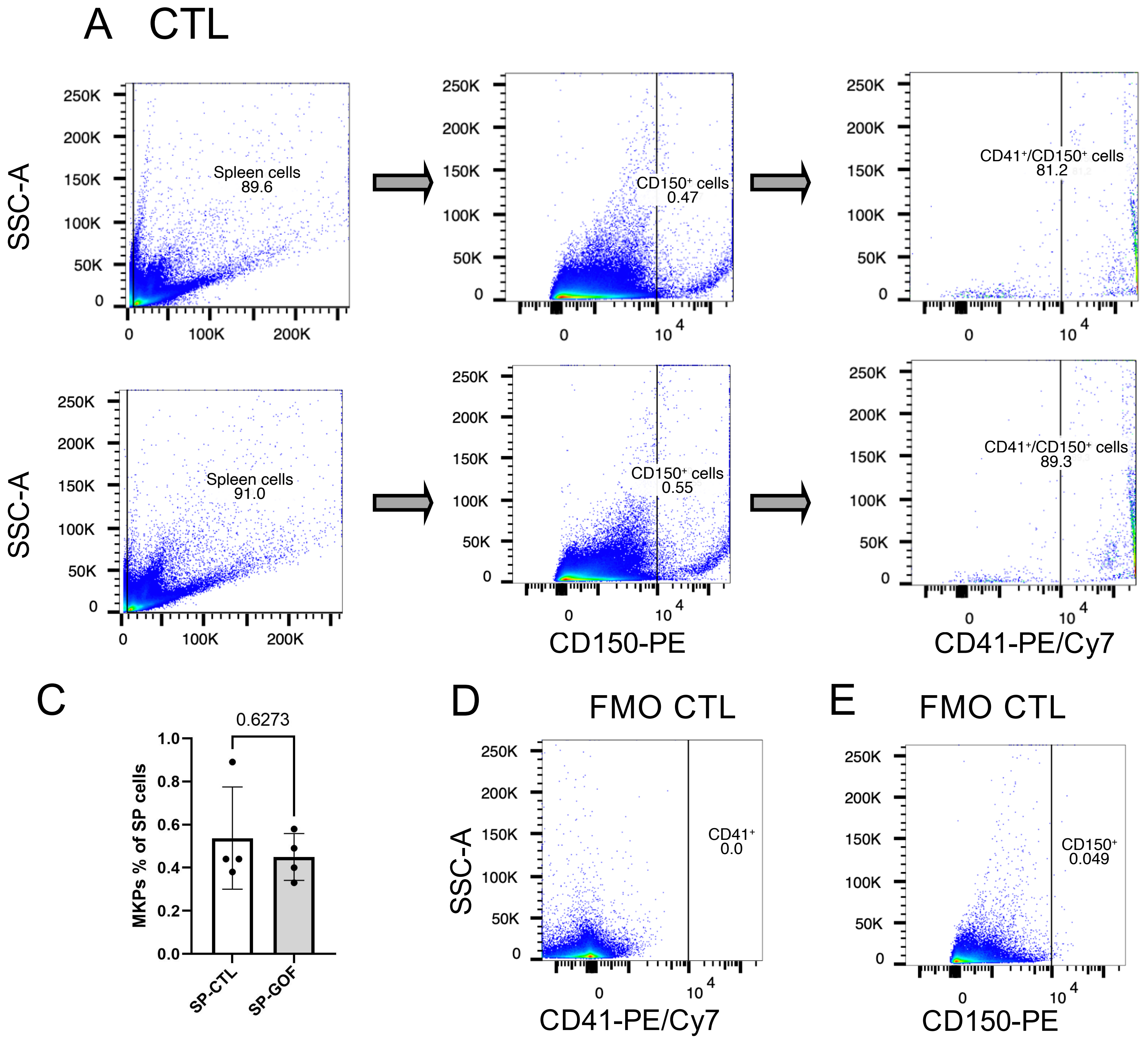

Suppl. Figure 12, Yalcin et al.

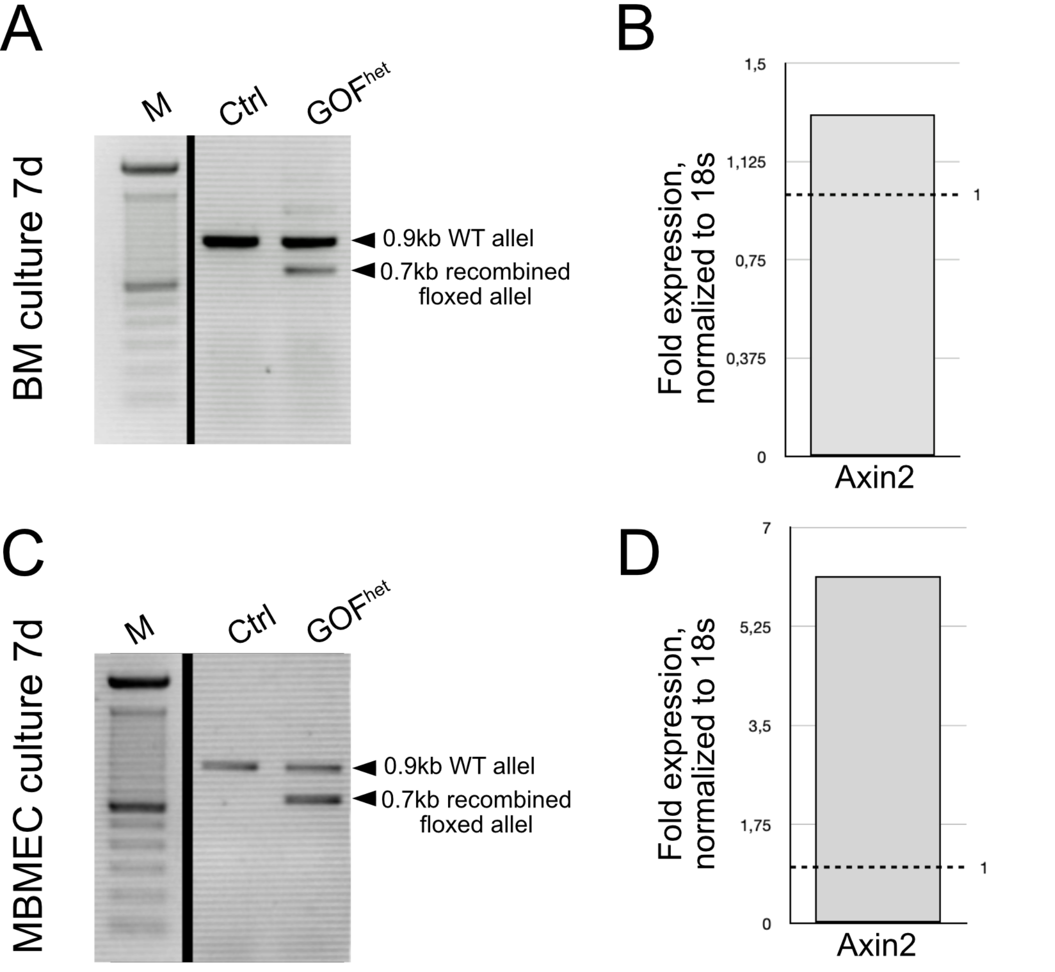

Supplement: Supplementary file 1 [file cells-12-02765-s001.zip › cells-2632484-supplementary.pdf]
